# The Factors Affecting Older Adults’ Intention toward Ongoing Participation in Virtual Reality Leisure Activities

**DOI:** 10.3390/ijerph16030333

**Published:** 2019-01-25

**Authors:** Tsu-Ming Yeh, Fan-Yun Pai, Mei-Yuan Jeng

**Affiliations:** 1 Department of Industrial Engineering and Management, National Quemoy University, Kinmen 892, Taiwan; tmyeh@nqu.edu.tw; 2 Department of Business Administration, National Changhua University of Education, Changhua 500, Taiwan; 3 Department of Leisure Recreation and Management Da-Yeh University, Changhua 515, Taiwan

**Keywords:** virtual reality, leisure activity, ongoing participation, experience values

## Abstract

Due to the aging of organs, older adults may have limited physical strength for participating in outdoor activities. Therefore, indoor activities offer an alternative for maintaining the health of older adults. Following advances in technology, individuals can use virtual reality to exercise in their homes and are no longer subject to the constraints of the outdoor environment or weather conditions. In addition, these activities are easier to participate in when compared to real-world leisure activities. The present research included 294 older adults as its research subjects. They were given firsthand experience of Wii games for 10 weeks, in order to examine the ongoing participation intention of older adults following an experience with virtual reality leisure activities. The study found that experience attributes, experience consequences, and experience values were important factors in determining ongoing participation intention and can effectively predict ongoing participation intention. Four experience attributes—ease of use, usefulness, safety and flexibility, and fun—significantly influenced the experience value and experience consequences of older adults’ participants. Experience values also influenced ongoing participation intention.

## 1. Introduction

Participation in leisure activities can help to prevent chronic diseases, reduce morbidity, and shorten the course of diseases in older adults, thereby reducing medical expenses and lowering the costs to society [1]. The more types of leisure activity that older adults participate in, the greater leisure benefits they can attain [2], increasing self-esteem and producing a positive effect through decreased anxiety and the relief of stress [3]. However, with advancing age, older adults have greater difficulty participating in sports than young people do, due to the decline in physiological functions and reduced mobility [4]. This produces a significant decline in rates of participation in sports and physical activities [5]. In addition, half of the individuals who participate in fitness activities will withdraw in a short period of time [6]. For older adults living in cities, rising house prices and increased demand for land caused by overcrowding have led to substantial reductions in urban green spaces, with large-scale recreational sports facilities being pushed to the margins of cities [7]. Therefore, leisure environment, crowded locations, convenience, and accessibility act as impediments to the participation of older adults. Through the use of technology, individuals can use virtual reality to exercise in their homes, where they are no longer subject to the constraints of the outdoor environment or weather conditions and can experience the pleasures of leisure activity indoors. Research by Saposnik et al. [8] has shown that the somatosensory Wii game console is a safe, and particularly suitable way for older adults to participate in leisure and fitness activities.

The Wii is seen as a health-promoting tool because it can provide purposeful and meaningful activities [9]. Also, it is a training tool for older adults [9]. The Wii was found to be a good way to make exercise more fun. In this case, the Wii was also used to improve balance. In addition, the Wii was used for dysfunctional people, such as for stroke rehabilitation or patients who are trying to improve their functional abilities [10]. To conclude, the majority of articles regarding the Wii and older adults have focused on the physical effects of using the Wii [9,10]. Only a few articles have explored why older adults are willing to use the Wii, and how to encourage older adults to use the Wii continuously. The current research regarding the Wii has focused on the physical effects and staff perceptions of implementing the Wii, but not on older adults’ own perspectives.

However, older adults are often not willing to accept new technology, often rejecting it completely due to the psychological pressure caused by the fear that they will not be able to use it correctly. The present study explores factors affecting the intention toward ongoing participation in virtual reality leisure activities among older adults. The outcomes of this study can help us to understand the psychological behavior of older people. Aside from helping the virtual reality software industry forecast the intentions of older consumers, the study results also provide the government and relevant units with a reference for the future promotion of another important leisure activity option for older adults, increasing their quality of life and improving their health.

## 2. Literature Review

### 2.1. Virtual Reality Leisure Activities

Virtual reality leisure activities refer to a computer-generated reality that allows users to enter a virtual world through a computer interface. In recent years, due to the rapid development of computer hardware and software, the computer animations generated by virtual reality technology have achieved an almost lifelike level of realism. In particular, the Wii Sports virtual reality sports games have transcended traditional video games. In 2008, Nintendo launched a new sports game, Wii Fit, featuring 21 different types of training. Each game type had its own sports training purpose, including balance training, aerobics, yoga, and muscle training, enabling users to better understand their own physical condition and providing health management benefits [11]. Wii sport is simple, safe, and fun, while providing players with the opportunity to exercise, making it a new option for sport, exercise, and weight loss. The Wii is perceived to be easy to use, providing a way for older adults to socialize with others, and giving them opportunities to participate in activities in new ways [8]. For older adults, especially those with disabilities, virtual reality leisure activities are easier to participate in as compared with real-world leisure activities [12].

Farrow and Reid [12] explored 16 stroke survivors’ perceptions of leisure-based virtual reality activities, finding that after participating in these activities the stroke survivors showed a significant increase in flow experiences and leisure competence. Wollersheim [13] also found that older adults have a relatively high level of interest in sports-based virtual reality leisure activities as compared to their interest in real-world leisure activities. Therefore, they are better able to gain enjoyment through their participation. Pigford and Andrews [14] found that when the Wii was used for rehabilitation, older adults were more willing to engage in rehabilitation and were more strongly motivated to participate in activities. Williams et al. [15] carried out a study of 21 older adults aged 76 and over in a senior living community. The study found that games improved the balance and self-confidence of older-adult community residents. Chiang et al. [16] conducted an experiment investigating the use of Wii Sports and Wii Fit among 58 older-adult subjects, showing that Wii Sports and Wii Fit not only improved balance but also led to a significant difference in postural stability. Bateni [17] divided 18 older-adult respondents equally into a traditional physical therapy group and a Wii Fit therapy group. The results showed that the group that combined traditional physical therapy with Wii Fit therapy achieved the greatest improvements in balance. These results provide empirical evidence for the health promotion benefits of participation in virtual reality leisure activities for older adults.

### 2.2. Experience

Experience is the subjective ideology of consumers, representing consumers’ perceptions and preferences toward product attributes and service performance. During the experience process, the tangible product value or service and intangible emotional value go beyond customers’ expected value. Customers are immersed in the process of consumption, which leaves a deep impression of the experience in their minds [18]. In the experience economy, it is believed that “feelings” can be sold and have higher added value than products [19]. For example, when visiting a play area, we are not purchasing a product or a service, but experiencing a feeling of fun and excitement. This shows that the experience economy has become a new source of value, affecting consumption decisions. Consumption is actually a process, and when the process is over, the memory will persist. From the perspective of Maslow’s hierarchy of needs, the experience of customization can help customers reach “self-actualization”. Therefore, development of products and technology seeks to satisfy the feelings and responses of consumers after they use the products.

### 2.3. Values

Values are an important predictor variable for consumer behavior, and are regarded as a better tool than customer satisfaction to ensure market share under diverse market competition [20], as they exert significant influence over consumer behavior. Some studies have examined the effects of personal values on the decision-making aspect of human behavior [21]. Woodruff [22] believed that values are the goals and objectives that customers seek to achieve through the process of consumption, and that they also influence assessments and cognitive preferences for product attributes and the performance and consequences of attributes. From an experience perspective, Holbrook [23] argued that consumer values are a type of preference that is influenced by individual likes and interests. Consumer values include self-esteem and affection and are used to communicate the attributes of a product or service to a consumer [24]. Therefore, when consumers evaluate products or services, values become an important factor. Behavior is only meaningful when the product features or attributes can satisfy certain basic needs or values. Incorporating personal values enables accurate predictions of consumer behavior [25].

### 2.4. Behavior Intention

Behavior intention refers to a customers’ views of the service quality generated by their actual feelings after experiencing a service. This produces a subjective judgment regarding the possibility of taking further actions, such as recommending the service to friends and family [26]. In the era of the experience economy, product sellers pay attention to the feelings and feedback of customers after they experience a product. Post-experience behavior intention affects ongoing participation intention and purchase intention. These are both important variables for predicting behavior and are related to post-experience satisfaction [19]. Rejikumar and Ravindran [27] asserted that ongoing intention to use a product is entirely dependent on the customer’s satisfaction with the product. Akter et al. [28] found that service quality and satisfaction can predict ongoing intention to use a product. A higher service quality produces greater satisfaction and, therefore, stronger ongoing intention to use the product. Zhao et al. [29] pointed out that service quality and fairness both affect the level of satisfaction, which affects ongoing intention to use a product. There is currently a lack of research on the behavioral intentions of older adults towards participation in virtual reality leisure activities after experiencing such participation. At present, some studies have shown that older adults with ongoing regular exercise habits enjoy significantly better health [30].

### 2.5. Research Hypotheses

Product attributes refer to the views of consumers towards a product. Consumers regard every product as a collection of attributes [31]. When experiencing or using products, consumers are typically interested in product attributes that can bring them benefits or solve specific problems. Even though certain product attributes or a need for the same type of product may differ between individual consumers, Strahilevitz and Myer [32] have argued that the functional attributes of products can satisfy the functional requirements of consumers and achieve customers’ problem-solving objectives—or enable consumers to achieve a certain function or task. This means that consumers typically assess product attributes before purchasing products to determine whether the products meet their values. Yu-Ling Lin and Hong-Wen Lin [33] pointed out that the attributes of virtual reality products should include their role, interface design, multiplayer games, independent games, popularity, and virtual pets. Kotler [31] also argued that product attributes affect consumers’ judgment of the product’s value. Therefore, we propose Hypothesis 1.

**Hypothesis** **1.** *The attributes of the experience of virtual reality leisure activities by older adults have a significant influence on their perception of its value*.

Consequences are the actual psychological or other benefits obtained by consumers during the experience process. When the result obtained is positive, it is called a benefit [34], but when it is negative, it is called a perceived risk. The benefits of virtual reality games to users are increased interaction, increased enjoyment, improved efficiency, novelty, and easing of stress [33]. Holbrook [23] argued that when choosing products, aside from considering their own product needs, consumers assess the value of a product by the image and symbolism that they impose onto it. That is, the result of the consumer experience is used to assess whether the ultimate objectives are reached. For example, visitors to green spaces might feel “physical well-being” or the sensation of having “helped the environment” (consequence) after the visit. The result here is an increase in the individual’s “quality of life”, with an ultimate value of obtaining a “happier life” [35]. Therefore, we propose Hypothesis 2.

**Hypothesis** **2.** *The consequences of the experience of virtual reality leisure activities by older adults have a significant influence on their perception of its value*.

Kim and Lee [36] stressed that personal values, preferences, and motivations can produce potentially massive changes in behavior. Experience quality, experience values, and level of satisfaction have been shown to be good predictors of behavior intention [37]. With regard to the effects of values on ongoing participation intention, Zhao et al. [29] and Akter et al. [28] asserted that values have a positive effect on ongoing participation intention. In addition, Rejikumar and Ravindran [27] showed that there is a close relationship between service quality and level of satisfaction and intentions for ongoing use. The intended ongoing use of products by consumers is entirely dependent on their level of satisfaction with the products. Most studies have shown that values have a positive influence on consumers’ ongoing behavior intentions. Therefore, we propose Hypothesis 3.

**Hypothesis** **3.** *The values of the experience of virtual reality leisure activities by older adults have a significant influence on ongoing participation intention*.

Schmitt [38] believed that aside from shaping a better consumer experience, experience models should also emphasize post-experience behavior. The result of the experience is the key factor determining customer satisfaction and brand loyalty. Consumer satisfaction is an active and emotional response caused by a favorable assessment of a shopping or consumer experience. In other words, satisfaction comes from the feeling by consumers that the product exceeded expectations, producing a positive influence on post-experience behavior [39,40]. Ongoing participation is determined by the strength of the relationship when the consumer triggers a particular behavior. When consumers perceive that product attributes provide important benefits, or that they have a close relationship with a product, ongoing participation intentions will increase [27]. Therefore, when consumers have a high ongoing participation intention towards a product, they may be interested in accumulating knowledge about the product and may be very sensitive to product attributes. In particular, when others mention the product, they will put forward their own views on the product, which will influence their own ongoing behavior intentions or those of others. Therefore, we propose Hypotheses 4 and 5.

**Hypothesis** **4.** *The attributes of the experience of virtual reality leisure activities by older adults have a significant influence on ongoing participation intention*.

**Hypothesis** **5.** *The consequences of the experience of virtual reality leisure activities by older adults have a significant influence on ongoing participation intention*.

Jeng and Yeh [41] claimed that after using the “attributes” of a product or service, consumers will evaluate its “consequences”. In a study of the value of urban green spaces, López-Mosquera and Sánchez [35] discovered that users believe that green spaces provide a “space to practice sports” (attribute). This attribute produces “better mental wellbeing” (consequence). The “distance from home” attribute produces the “regular user of green space” consequence. In addition, users believe that the “benefits to health” attribute can produce “stress relief” consequences. This shows that experience attributes have a positive influence on experience consequences. Therefore, we propose Hypothesis 6.

**Hypothesis** **6.** *The attributes of the experience of virtual reality leisure activities by older adults have a significant influence on consequences*.

**Figure 1:**
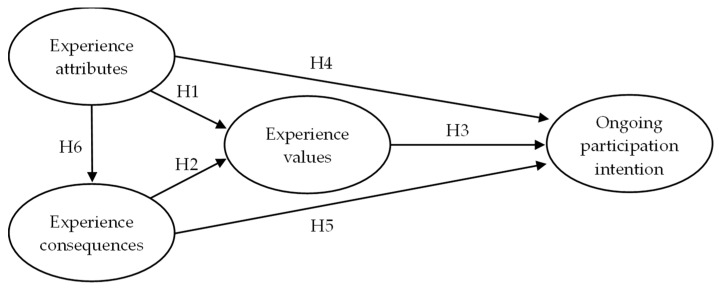
Research framework.

Based on the existing literature, the present study proposed six hypotheses on the relationships between attributes, consequences, values, and behavioral intentions for virtual reality leisure activities, developing a behavioral intentions model for older consumers’ experiences of virtual reality leisure activities, as shown in Figure 1.

## 3. Research Methods

### 3.1. Measurement Tools

In order to understand the perceptions of attributes, experience consequences, and post-experience values of older-adult participants in virtual reality leisure activities, and to forecast subsequent ongoing participation intention, we adopted a structured questionnaire with five main parts. The first part was social and demographic variables, including age, sex, education level, current living conditions, and monthly disposable income. The second to the fifth parts included measures of experience attributes, experience consequences, experience values, and ongoing participation intention, respectively. Aside from the demographic variables in the first part, a Likert five-point scale was used for the remaining four measures to measure respondents’ levels of agreement with each of the descriptions, from “strongly disagree” (1) to “strongly agree” (5). After the preliminary design of the questionnaire, we sent the questionnaire and procedure to the Research Ethics Committee of National Cheng Kung University, Taiwan for ethical review. This study was conducted under approval number No. 104-154.

### 3.2. Data Collection

The paper-based written consent form is typically the easiest, fastest, and most cost-effective means to document the terms of consent and to permit consent traceability [42]. We provided paper-based informed consent details on the objectives and methods, as well as the potential risks and benefits of the research, to ensure that all participants were able to give informed consent before we asked the older adults to fill out the questionnaire.

Data were collected in two stages. First, in order to select older-adult respondents with “real” experiences of using the Wii, we chose eight high-quality older-adult community care centers and invited older-adult respondents who were willing to try the Wii. In total, we recruited 320 respondents who had not previously used the Wii and then provided a Wii device for free to each center, and used the care center activity times each Monday to Friday to dispatch staff who would set up the Wii and teach respondents how to use it. Each respondent had an average of 10 opportunities to play the various games offered on the Wii, with 2 to 4 people playing each game at the same time, with a new group taking over when the original group became tired. In the second stage, after 10 weeks, 8 interviewers visited the communities to interview the respondents who had experienced the Wii. Taking vision and ability to complete the form into consideration, interviewers read the questionnaire aloud to respondents in one-to-one sessions. The respondents each selected one of five responses (“strongly agree,” “agree,” “neither agree nor disagree,” “disagree”, or “strongly disagree”) based on their post-experience feelings. Some of the respondents withdrew during the survey. There were 294 valid questionnaires, giving an actual response rate of 92%.

### 3.3. Operational Definitions

The term “experience attributes” refers to older-adult respondents’ views of product attributes during the process of experiencing the Wii. Virtual reality leisure activities are an aspect of information technology products. The questionnaire design for attributes was based on the dimensions identified by Davis [43] and Van der Heijden [44], including ease of use, usefulness, safety and flexibility, and fun. We developed 10 question items based on these ideas.

The term “experience consequences” focuses on the psychological benefits obtained by older adults, such as sensory excitement or inner needs, following their participation in virtual reality activities. Therefore, we used the four types of individual experience identified by Pine II and Gilmore [19]—entertainment, aesthetics, education, and escapism—as the four dimensions of the scale. Based on these results, we developed 10 question items. “Experience values” means satisfying individual values through the possession of a product or the experience process. In this study, we referred to the list of values (LOV) scales of Jeng and Yeh [41] and Kahle and Kennedy [45] to develop three questions.

The term “ongoing participation intention” refers to the probability or possibility that consumers will continue to participate in virtual reality leisure activities in the future. The scale is based on Hus and Lu’s [46] study of ongoing participation in online games. The scale contains two items: “I will continue to play Wii in the future” and “there is a very high probability that I will continue to play Wii in the future”. Questionnaire items are demonstrated in the Appendix (Table A1).

## 4. Research Results

### 4.1. Analysis of the Sample

In total, 294 valid samples were gathered. The proportions of male and female respondents were 55.1% and 44.9%, respectively. Of the respondents, 50% had high school or higher education. The largest number of respondents had a monthly income in the range of NT$20,001 to NT$35,000 (accounting for 34.7%), followed by an income between NT$35,001 and NT$50,000 (accounting for 33.3%). A total of 50.3% of the respondents were aged between 66 and 70, while 78.9% of the respondents were married. In terms of housing situation, the greatest number of respondents lived with family members, accounting for 75.2% of the total, followed by those living with their spouse, who made up 22.1% of the respondents. Overall, the largest number of respondents were male, aged between 66 and 70, had a high school or higher education, lived with family members, and had a monthly income in the range of NT$20,001 to NT$35,000. Table 1 shows the socio-demographic profile of the respondents.

The structure equation model was then used to verify the proposed hypotheses. IBM SPSS Amos was used for data analysis.

### 4.2. Measurement Model

#### 4.2.1. Correlation Analysis

Related endogenous variables included experience attributes (A1–A4), experience consequences (C1–C4), experience values (V1–V3), and ongoing participation intention (R1, R2). The significance level of all variables was (*p* < 0.05). Although there was no limitation in terms of high correlations, we would have been very cautious if the coefficient had exceeded 0.90. In many cases, a correlation coefficient exceeding 0.80 indicates a problem with multicollinearity. The correlations between the 13 variables in this study ranged from 0.44 to 0.79, indicating no problems with collinearity. All of the variables reached the significance level (*p* < 0.01), showing good correlation between variables [47], as shown in Table 2.

#### 4.2.2. Evaluation of the Model’s Goodness of Fit

Bagozzi and Yi [48] suggested that the sample size should be based on the model fit, measured as the ratio of the chi-square value (χ^2^) to its degrees of freedom. However, the chi-square value is very sensitive to the size of the sample being tested. With a larger sample size, it is easier for the chi-square value to reach significance, increasing the possibility of the theoretical model being rejected. Therefore, the normed chi-square (normedχ^2^) (χ^2^/df) has been suggested as a better measure of the goodness-of-fit ratio. In order to test the model–fit ratio, the present study used the chi-square degree of freedom ratio (χ^2^/df). A ratio of less than 3 is an acceptable model–fit ratio. The overall goodness-of-fit indices were χ^2^/df = 2.28, GFI = 0.93, AGFI = 0.90, CFI = 0.97, RMR = 0.01, and PGFI = 0.76 (see Table 3). In addition, the AIC of the default model (129.997) was smaller than the AIC values of both the saturated model (166.223) and the independent model (1782.661). These results show that all of the variables met the criteria for validating the research hypotheses.

#### 4.2.3. Convergent Validity Analysis

In this study, the factor-loading coefficients for the measured variables were in the range of 0.70 to 0.92, and the factor-loading t-value was in the range of 13.45 to 20.44, showing that the measured variables all reached the significance level. In terms of composite reliability, the estimates of the composite reliability of the latent variables, including experience attributes, experience consequences, experience values, and ongoing participation intentions, were 0.90, 0.88, 0.84, and 0.73, respectively, reaching a standard of 0.7 or higher, indicating that the model has intrinsic quality. In terms of the average variance extracted (AVE), the results showed that the AVE was 0.70 for experience attributes, 0.66 for experience consequences, 0.64 for experience values, and 0.58 for ongoing participation intention. The AVE for each of the variables reached the threshold of 0.5 proposed by Fornell and Larcker [49], and the factor loading of each of the questionnaire items had a very high level of significance (*p* < 0.001), showing that the observed variables were sufficient for reflecting the constructed latent variables. Therefore, the measurement model has good convergence validity, as shown in Table 4.

### 4.3. Hypothesis Testing

We carried out a structural model test based on the results of the confirmatory factor analysis (CFA). From the goodness-of-fit statistics in the overall model, we found that the structure of the model was acceptable (χ^2^/df = 2.28, GFI = 0.93, AGFI = 0.89, CFI = 0.97, RMR = 0.02). Therefore, it was not necessary to revise the model. Next, we used maximum likelihood to estimate the path coefficient values for each of the latent variables. The six research hypotheses are shown in Table 5.

The parameter estimates and test results in Table 5 show that all six hypotheses were supported.

**Hypothesis** **1.** The experience attributes of virtual reality leisure activities have a positive influence on experience values—supported (*β* = 0.51; *t* = 4.54; *p* < 0.001).

**Hypothesis** **2.** The experience consequences of virtual reality leisure activities have a positive influence on experience values—also supported (*β* = 0.36; *t* = 3.68; *p* < 0.001). Experience consequences have a significant influence on experience values. The results confirmed that experience attributes and experience consequences both have positive influences on experience values, and the influence of experience attributes on experience values is stronger.

**Hypothesis** **3.** The experience values of virtual reality leisure activities have a positive influence on ongoing participation intention—also supported (*β* = 0.59; *t* = 9.67; *p* < 0.001).

**Hypothesis** **4.** The experience attributes of virtual reality leisure activities have a positive influence on ongoing participation intention—also supported (*β* = 0.16; *t* = 2.50; *p* < 0.01).

**Hypothesis** **5.** The experience consequences of virtual reality leisure activities have a positive influence on ongoing participation intention—also supported (*β* = 0.14; *t* = 2.16; *p* < 0.05).

**Hypothesis** **6.** The experience attributes of virtual reality leisure activities have a positive influence on experience consequences—also supported (*β* = 0.79; *t* = 22.51; *p* < 0.001).

**Figure 2:**
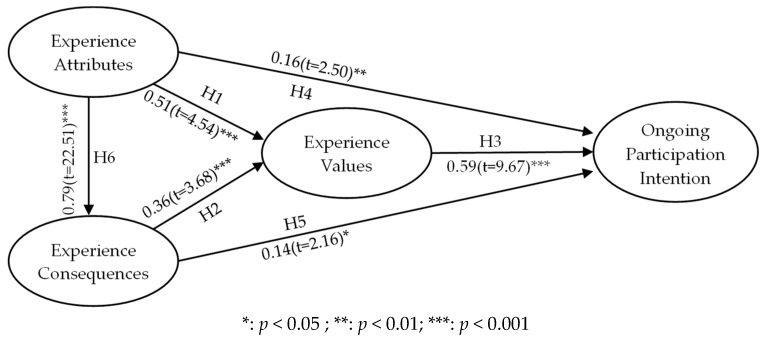
Path results for the behavior intentions of older adult participants.

In short, experience attributes and experience consequences were determined to have positive influences on experience values. Experience values as well as experience attributes and experience consequences also influenced ongoing participation intention, while experience attributes also influenced experience consequences (see Figure 2).

## 5. Conclusions

The emergence of virtual reality leisure activities has provided a new activity choices for older adults. Through interaction in games, older adults can maintain close relationships with others, encouraging their sense of worth, which is very important for helping older adults participate in society. The results of the study found that experience attributes, experience consequences, and experience values are important factors that determine ongoing participation intention. As long as older adults are encouraged to believe that virtual reality leisure activities have health promotion benefits, they will generate ongoing participation intention. In order to make older adults willing to participate in virtual reality leisure activities, firms or other organizations should study the needs and expectations of older adults toward virtual reality leisure activities in detail. They could enable older adults to actually try out the activities, thereby facilitating the older adults’ accumulation of experience, or provide personalized consultation services to reduce the rejection of technology by older-adult consumers.

For older adults living in nursing homes, fun can be used to trigger the intrinsic and extrinsic motivation of residents. Using games to encourage participation in activities to promote health is essential. At present, some experts in physical therapy and recreational therapy have started to use virtual activities so that patients can move beyond the boring and monotonous rehabilitation methods in traditional medical treatment. When compared to expensive medical equipment that most people have difficulty accessing, cheap and lightweight virtual reality leisure activity software offers more practical value. Sound and light can stimulate the cognitive function of older-adult users, while interactive games promote joint motion. In addition, the immediate feedback interface increases self-confidence, and fun gaming experiences help to maintain dynamic living. These features make virtual activities suitable for use in care institutes, providing a new opportunity and direction for health promotion among older adults.

## Figures and Tables

**Table 1:** Socio-demographic profile of the respondents.

Category	Items	No.	%
Gender	male	162	55.1%
	female	132	44.9%
Age	61–65 years old	30	10.2%
	66–70 years old	148	50.3%
	71–75 years old	80	27.2%
	76 years old above	36	12.2%
Education level	elementary school	30	10.2%
	junior high school	12	4.1%
	senior high school	90	30.6%
	college or above	162	55.0%
Monthly income	NT$20,000 or less	46	15.6%
	NT$20,001–NT$35,000	102	34.7%
	NT$35,001–NT$50,000	98	33.3%
	NT$50,001–NT$65,000	26	8.6%
	NT$65,001 or above	22	7.5%
Housing situation	lived with family members	221	75.2%
	living with their spouse	65	22.1%
	living on their own	8	2.7%
Marital status	married	232	78.9%
	widowed	44	15.0%

**Table 2:** Correlations between variables.

Variable	A1	A2	A3	A4	C1	C2	C3	C4	V1	V2	V3	R1	R2
A1. Ease of use	1												
A2. Usefulness	0.79	1											
A3. Safety and flexibility	0.72	0.74	1										
A4. Fun	0.66	0.65	0.66	1									
C1. Entertainment experience	0.65	0.64	0.60	0.59	1								
C2. Aesthetic experience	0.60	0.68	0.61	0.51	0.70	1							
C3. Escapism experience	0.48	0.48	0.46	0.58	0.57	0.52	1						
C4. Education experience	0.72	0.71	0.68	0.70	0.70	0.74	0.68	1					
V1. Enjoyment of life	0.62	0.57	0.57	0.57	0.54	0.54	0.42	0.64	1				
V2. Sense of belonging	0.63	0.60	0.56	0.59	0.54	0.54	0.54	0.71	0.66	1			
V3. Good memories	0.78	0.68	0.64	0.60	0.58	0.62	0.51	0.68	0.62	0.63	1		
R1. Ongoing participation	0.59	0.55	0.56	0.50	0.53	0.54	0.44	0.60	0.57	0.60	0.61	1	
R2. May continue to play	0.60	0.57	0.57	0.53	0.53	0.55	0.40	0.61	0.58	0.68	0.66	0.57	1
Mean	4.14	4.39	4.19	4.21	4.11	4.19	3.55	4.30	4.10	3.98	4.14	4.11	4.21
Standard deviation	0.56	0.64	0.63	0.78	0.55	0.55	0.80	0.75	0.74	0.85	0.87	0.76	0.79

All variables are statistically significant (*p* < 0.01).

**Table 3:** Model’s goodness-of-fit indices.

Name of Index	Standard	Research Results
χ^2^/df	<3	2.28
GFI	>0.9	0.93
AGFI	>0.9	0.90
CFI	>0.9	0.97
RMR	<0.08	0.01
PGFI	>0.5	0.76
AIC		120.997

**Table 4:** Confirmatory factor analysis (CFA) of the variable measurement model.

Variable		Factor Loading	Reliability Coefficient	Measurement Error	*t*-Value	Degree of Combination	AVE
Experience attributes	A1	0.87	0.75	0.25	18.55 ***	0.90	0.70
	A2	0.87	0.73	0.25	18.41 ***		
	A3	0.83	0.69	0.31	17.27 ***		
	A4	0.78	0.61	0.39	15.52 ***		
Experience consequences	C1	0.79	0.62	0.38	16.03 ***	0.88	0.66
	C2	0.81	0.66	0.34	16.51 ***		
	C3	0.70	0.49	0.51	13.45 ***		
	C4	0.92	0.85	0.15	20.44 ***		
Experience values	V1	0.76	0.58	0.42	15.07 ***	0.84	0.64
	V2	0.81	0.66	0.34	16.43 ***		
	V3	0.82	0.67	0.33	16.72 ***		
Ongoing participation intention	R1	0.78	0.61	0.39	13.94 ***	0.73	0.58
	R2	0.74	0.55	0.45	14.82 ***		

***: *p* < 0.001.

**Table 5:** Estimates of the path coefficients in the research model.

Path	Path Coefficient	*t*-Value	Reliability Coefficient
H1 experience attributes → experience values	0.51	4.54 ***	0.26
H2 experience consequences → experience values	0.36	3.68 ***	0.13
H3 experience values → ongoing participation intention	0.59	9.67 ***	0.34
H4 experience attributes → ongoing participation intention	0.16	2.50 **	0.03
H5 experience consequences → ongoing participation intention	0.14	2.16 *	0.02
H6 experience attributes → experience consequences	0.79	22.51 ***	0.62

*: *p* < 0.05; **: *p* < 0.01; ***: *p* < 0.001.

## Appendix A

**Table A1:** Variable measurements.

Variables	Questions	Reference
Experience attributes	Ease of use 1. I can do exercise when I’d like to with Wii. 2. I can do exercise easily with Wii. 3. It’s easy and convenient to use Wii to do exercise.	Davis [41]; Van der Heijden [42]
	Usefulness 1. Exercising with Wii improves my psychological and physical health. 2. Using Wii improve my interactions with other people. 3. Using Wii is interesting.	
	Safety and flexibility 1. Exercising with Wii keeps my mind and actions quick. 2. It’s safe for me to use Wii.	
	Fun 1. Exercising with Wii is fun. 2. Wii is an amazing product.	
Experience consequences	Entertainment experience 1. Exercising with Wii make me feel relax. 2. Exercising with Wii make me feel happy.	Pine II and Gilmore [17]
	Aesthetic experience 1. Exercising with Wii satisfies my curiosity. 2. I can experience virtual-reality leisure activities by exercising with Wii. 3. I learn about innovative products by exercising with Wii.	
	Escapism experience 1. I feel like I have adventures when exercising with Wii. 2. Exercising with Wii is exciting.	
	Education experience 1. I can learn new knowledge when exercising with Wii with others. 2. I can learn new information when exercising with Wii with others. 3. I can share my life experience when exercising with Wii with others.	
Experience values	1. Exercising with Wii improves my enjoyment of life. 2. Exercising with Wii improves my sense of belonging. 3. Exercising with Wii creates good memories.	Jeng and Yeh [39]; Kahle and Kennedy [43]
Ongoing participation intention	1. I’ll continue to exercising with Wii. 2. I’ll invite my friends to exercise with me using Wii.	Hus and Lu [44]
